# Associação entre a Gravidade da Doença Arterial Coronariana e Câncer de Pulmão: Um Estudo Piloto Transversal

**DOI:** 10.36660/abc.20200478

**Published:** 2021-12-16

**Authors:** Mingzhuang Sun, Qian Yang, Meng Li, Jing Jing, Hao Zhou, Yundai Chen, Shunying Hu

**Affiliations:** 1 Chinese PLA General Hospital Department of Cardiology Beijing China Chinese PLA General Hospital - Department of Cardiology, Beijing – China; 2 Chinese PLA General Hospital Department of Medical Records Management Beijing China Chinese PLA General Hospital - Department of Medical Records Management, Beijing – China

**Keywords:** Doença da Artéria Coronariana/complicações, Neoplasias Pulmonares/complicações, Angiografia Coronária Percutânea/métodos, Índice de Gravidade de Doença, Escore Sintax, Intervenção Coronária Percutânea/métodos

## Abstract

**Fundamento:**

A relação direta entre a doença arterial coronariana (DAC) e o câncer de pulmão não é bem conhecida.

**Objetivo:**

Investigar a associação entre a gravidade anatômica da DAC e do câncer de pulmão.

**Métodos:**

Trezentos pacientes, incluindo 75 recém-diagnosticados com câncer de pulmão e 225 pacientes correspondentes sem câncer, foram submetidos à angiografia coronária durante a internação, sem intervenção coronária percutânea (ICP) prévia nem enxerto de bypass da artéria coronária (CABG). O escore SYNTAX foi utilizado para avaliar a gravidade da DAC. Uma pontuação alta no escore foi definida como > 15 (o maior quartil do escore SYNTAX). O teste de tendência de Cochran-Armitage foi utilizado para verificar a distribuição dos escores dos pacientes. Uma análise de regressão logística foi utilizada para avaliar a associação entre a gravidade da DAC e o câncer de pulmão. Os valores de p foram estabelecidos quando o nível de significância era 5%.

**Resultados:**

A tendência de distribuição dos escores SYNTAX dos pacientes por quartis foi diferente entre aqueles com câncer de pulmão e controles (do quartil mais baixo ao mais alto: 20,0%; 20,0%; 24,0%; 36,0% vs. 26,7%; 26,2%; 25,8%; 21,3%; p=0,022). A pontuação no escore SYNTAX foi mais alta em pacientes com câncer do que nos pacientes controle (36,0% vs. 21,3%, p=0,011).O maior quartil do escore demonstrou mais riscos de desenvolver câncer de pulmão em comparação ao quartil mais baixo (OR: 2.250, IC95%: 1.077 a 4.699 ; *P* -trend= 0,016). Após ajustes, os pacientes no maior quartil do escore SYNTAX tinham mais risco de desenvolver câncer de pulmão (OR: 2.1o49, IC95%: 1.008 a 4.584; *P* -trend= 0,028). Pacientes com escores SYNTAX alto (> 15) tinham 1.985 mais chances de ter câncer de pulmão (IC95%: 1.105–3.563, P= 0,022).

**Conclusão:**

A gravidade anatômica da DAC está associada ao risco de câncer de pulmão, o que indica que um rastreamento completo deste tipo de câncer possa ser mais significativo entre pacientes com DAC.

## Introdução

Doenças cardíacas e cânceres são problemas de saúde críticos para seres humanos em todo o mundo.^1 , 2^ A toxicidade cardiovascular induzida pela terapia anticâncer levou ao novo campo interdisciplinar da cardio-oncologia.^2 - 5^ No momento, oncologistas e cardiologistas estão mais focados em doenças cardíacas relacionadas à terapia anticâncer entre os sobreviventes de câncer.^6 , 7^ Alguns estudos demonstram que há uma relação direta de interação entre doenças cardíacas e câncer.^8^

A doença arterial coronariana (DAC) e o câncer compartilham fatores de risco em comum, além de mecanismos fisiopatológicos.^9 - 11^ Há grandes evidências que mostram que pacientes com câncer tinham mais riscos de desenvolver doenças cardiovasculares. Um estudo sueco mostrou que os cânceres estão associados ao maior risco de DAC; porém, neste caso, os pacientes tinham recebido tratamentos anticâncer, então o estudo não pôde demonstrar se havia ou não uma relação direta entre a DAC e o câncer.^2^ Este fato de a gravidade da CAD estar ou não associada ao câncer foi pouco reportado até o momento. É essencial esclarecer se há uma relação potencial direta entre a DAC e o câncer para um melhor entendimento e administração dessas duas doenças.

O câncer de pulmão é o tipo de câncer mais comum, e uma das principais causas de morte ligada ao câncer.^12^ Vale investigar a associação entre a DAC e o câncer de pulmão. Uma meta-análise mostrou que o câncer de pulmão esteve associado ao risco significativamente maior de DAC durante o acompanhamento em comparação aos pacientes sem câncer de pulmão.^13^ No estudo, os pacientes com câncer de pulmão foram acompanhados por mais de um ano após serem diagnosticados, então havia mais chances de terem recebido tratamentos anticâncer, o que não poderia excluir o efeito desses tratamentos na CAD. A relação direta entre a DAC e o câncer de pulmão não é bem compreendida até hoje. A hipótese de que a gravidade da CAD está associada a este tipo de câncer não foi reportada até o momento.

Realizamos um estudo transversal para investigar a associação direta entre a gravidade anatômica da CAD e o câncer de pulmão. Todos os pacientes com este tipo de câncer foram recentemente diagnosticados e não receberam nenhum tratamento anticâncer. Os pacientes controle não tinham câncer de pulmão e foram incluídos no estudo utilizando escores de propensão relacionados a pacientes com câncer. Todos os pacientes do estudo foram submetidos à angiografia coronária (AGC) durante a hospitalização inicial. A gravidade anatômica da DAC foi avaliada utilizando o escore SYNTAX, com base nas AGC.^14^ O objetivo deste estudo foi determinar se a gravidade anatômica da DAC está relacionada ao risco de câncer de pulmão.

## Pacientes e Métodos

### Pacientes do estudo

No Hospital Geral Chinese PLA, todos os dados dos pacientes internados estão armazenados no registro médico, incluindo os angiogramas coronários. Havia 173 pacientes com câncer de pulmão (CID-10, código 34) e 49.968 sem nenhum tipo de câncer que foram submetidos à AGC (CID-9-MC, códigos 88,5; 88,55; 88,56 e 88,57) no departamento de cardiologia, de 1º de janeiro de 2009 a 31 de julho de 2019.

**Figura 1:**
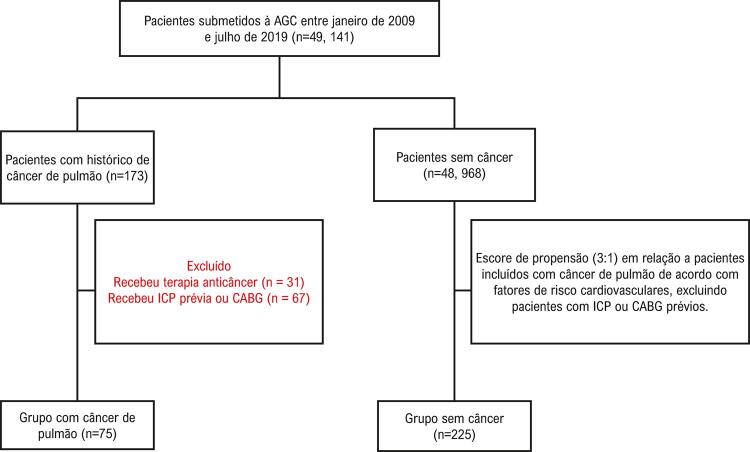
– Fluxograma dos pacientes do estudo.

Os diagnósticos de câncer de pulmão foram validados de acordo com o diagnóstico patológico. Dos 173 pacientes com câncer de pulmão, 75 tinham sido recém-diagnosticados sem receber nenhum tratamento anticâncer e foram inseridos no estudo, à exceção de 31 com histórico de tratamento anticâncer e 67 que já tinham sido submetidos à intervenção coronária percutânea (ICP) ou enxerto de bypass da artéria coronária (CABG). Dos 48.968 pacientes sem câncer, 225 foram incluídos como pacientes controle por meio de escores de propensão em relação aos indivíduos com câncer (razão de 1:3), de acordo com idade, gênero, histórico familiar de DAC, índice de massa corporal (IMC), hábito de fumar, hipertensão, diabetes e hiperlipidemia. Nenhum dos pacientes incluídos tinha histórico de doença do tecido conjuntivo ou outras doenças inflamatórias, nem de ICP ou CABG antes da hospitalização inicial. Um fluxograma do processo de inclusão dos pacientes é demonstrado na Figura 1 .

Calculamos o tamanho da amostra utilizando o programa PASS. De acordo com parâmetros incluindo a = 0,05; β = 0,20; OR= 2 e a razão do caso-controle = 1:3, o tamanho da amostra do grupo com câncer de pulmão foi de 74, e o do grupo dos indivíduos sem câncer foi de 222. Neste estudo, incluímos 300 pacientes (75 com câncer de pulmão e 225 sem câncer) que estavam de acordo com a exigência do tamanho da amostra.

### Escores SYNTAX da CAD com base na AGC

A gravidade da CAD foi avaliada utilizando o algoritmo do escore SYNTAX (descrito na íntegra em outros estudos).^14 , 15^ Todas as variáveis angiográficas relacionadas ao cálculo do escore SYNTAX foram computadas por dois cardiologistas intervencionistas experientes e cegos. Quando o escore de cada paciente foi diferente entre os dois cardiologistas, eles discutiam os angiogramas e chegavam a um escore comum. Os escores SYNTAX finais foram calculados por paciente e salvos em uma base de dados dedicada. O escore de 15 foi o maior quartil do estudo. Um escore baixo foi definido como ≤15, e um escore alto, como >15. Na análise de regressão logística, definimos o escore >15 como positivo.

### Análise estatística

As estatísticas descritivas estão apresentadas como taxas de frequência e porcentagem para variáveis categóricas, e média ± desvio padrão (DP) e medianas [intervalo interquartil (IIQ)] para variáveis contínuas, de acordo com a normalidade dos dados. Avaliamos a normalidade dos dados com os testes de normalidade de Skewness e Kurtosis. Utilizamos o teste t para amostras independentes para comparar as médias entre os grupos quando as variáveis foram distribuídas normalmente. O teste U de Mann-Whitney não-paramétrico foi usado para as variáveis contínuas com distribuição normal, incluindo triglicérides, lipoproteína de baixa densidade, lipoproteína de alta densidade, glicemia de jejum, alanina aminotransferase, aspartato aminotransferase, creatinina sérica e ácido úrico. Os testes qui-quadrado ou exato de Fisher foram usados para analisar as diferenças nas medidas categóricas.

O escore SYNTAX foi dividido em quartis com base na distribuição do escore em todos os indivíduos do estudo, sendo que o primeiro quartil foi usado como referência. Os valores do escore SYNTAX para os quartis 1, 2, 3 e 4 foram *<* 4,0; 4,0–9,0; 9,0–15,0 e *>* 15,0, respectivamente. O teste de tendência de Cochran-Armitage foi utilizado para analisar a distribuição dos escores dos pacientes. Avaliamos a relação entre a gravidade da DAC (escore SYNTAX estratificado em quartis) e o câncer de pulmão utilizando a análise de regressão logística, ajustando para fatores de risco comuns, incluindo idade, IMC, gênero e o hábito de fumar. A razão de chances (OR) e os intervalos de confiança de 95% (IC) foram calculados. Os valores de p eram bicaudais, e consideramos como significativos quando 5%. As análises estatísticas foram realizadas com o software SPSS, versão 23 (SPSS, Inc. Chicago, IL, EUA) e com o SAS (SAS Institute, Cary, Carolina do Norte).

## Resultados

### Características dos pacientes

Os indivíduos do estudo faziam parte de uma população específica do hospital geral Chinese PLA. A idade média dos 300 pacientes do estudo era 63,5±9,7 anos, e 70 (23,3%) eram do sexo feminino. Todos os 75 pacientes com câncer de pulmão tinham evidências patológicas da doença; 69 (92%) foram diagnosticados com câncer de pulmão de células não pequenas; e os outros 6 (8%), com câncer de pulmão de células pequenas. Quarenta e oito pacientes (75%) tinham câncer estágio I ou II; 12 deles (16%), estágio III; e 4 (5,3%), estágio IV; não foi possível confirmar o estágio da doença para outros 11 pacientes (14,7%). Quatro pacientes (5,3%) apresentavam metástase. Os pacientes com câncer de pulmão não tinham recebido tratamentos anticâncer antes da hospitalização inicial.

Não houve diferença significativa para idade, gênero, hábito de fumar (estratificado como os que nunca fumaram e os que sempre fumaram), IMC, histórico familiar de DAC, hipertensão, diabetes e hiperlipidemia entre pacientes com câncer de pulmão e pacientes sem câncer. Porém, o histórico de fumar apresentou diferenças entre o grupo com câncer de pulmão e os pacientes controle quando o histórico de fumar foi estratificado como os que nunca fumaram, os que fumaram no passado e os que fumam atualmente, o que foi ajustado na análise de regressão logística. A fração de ejeção (FE) e os dados laboratoriais foram comparáveis entre os pacientes com câncer de pulmão e os controle ( Tabela 1 ).

**Tabela 1:** – Características dos pacientes

Característica	Grupo câncer de pulmão (n = 75)	Grupo sem câncer (n = 225)	Valor de p
**Gênero, n (%)** Masculino Feminino	59 (78,7%) 16 (21,3%)	171 (76,0%) 54 (24,0%)	0,636^a^
**Idade (Anos)** <65 ≥65	34(45,3%) 41(54,7%)	120 (53,3%) 105 (46,7%)	0,230^a^
**IMC, n (%)** ≤24 >24	25 (33,30%) 50 (66,70%)	73 (32,4%) 152 (67,6%)	0,887^a^
**Fumar, n (%)** Nunca fumaram Sempre fumaram	27(36,0%) 48(64,0%)	97 (43,1%) 128 (56,9%)	0,279^a^
**Fumar, n (%)** Nunca fumaram Já fumaram Fumam atualmente	27 (36,0%) 21 (28,0%) 27 (36,0%)	97 (43,1%) 30 (13,3%) 98 (43,6%)	0,014^a^
**Histórico familiar de DAC, n (%)** Não Sim	69 (92,00%) 6 (8,00%)	196 (87,1%) 29 (12,9%)	0,253^a^
**Hipertensão, n (%)** Não Sim	29 (38,7%) 46 (61,3%)	84 (37,3%) 141 (62,7%)	0,836^a^
**Diabetes, n (%)** Não Sim	40 (53,3%) 35 (46,7%)	131 (58,2%) 94 (41,8%)	0,459^a^
**Hiperlipidemia, n (%)** Não Sim	25(33,3%) 50(66,7%)	95 (42,2%) 130 (57,8%)	0,174^a^
**Fração de Ejeção (%), n (%)** <50 ≥50	5(7,0%) 66(93,0%)	21 (11,7%) 159 (88,3%)	0,279^a^
**Dados laboratoriais** Colesterol total Triglicérides, mmol/L LDL-C , mmol/L HDL-C, mmol/L Glicemia de jejum, mmol/L ALT, U/L AST, U/L Albumina, g/L Creatinina sérica, µmol/L Ácido úrico, mmol/L Hemoglobina, g/L	4,035±0,950 1,420 (IIQ [1,055–1,780]) 2,545 (IIQ [1,838–3,195]) 1,070 (IIQ [0,833–1,265]) 5,820 (IIQ [4,980–7,720]) 20,50 (IIQ [11,70–27,20]) 17,20 (IIQ [13,70–25,10]) 40,589±4,056 76,70 (IIQ [66,80–84,80]) 328,10 (IIQ [266,00–396,70]) 135,76±16,647	4,029±1,015 1,360 (IIQ [1,020–1,860]) 2,340 (IIQ [1,850–3,058]) 0,980 (IIQ [0,830–1185]) 5,710 (IIQ [4,895–7,725]) 21,40 (IIQ [15,20–30,70]) 18,35 (IIQ [14,70–24,55]) 40,640±6,459 75,50 (IIQ [64,90–89,25]) 324,95 (IIQ [268,28–358,13]) 139,55±16,059	0,968^b^ 0,854^c^ 0,497^c^ 0,096^c^ 0,598^c^ 0,049^c^ 0,355^c^ 0,949^b^ 0.910^c^ 0,984^c^ 0,088^b^
**Escore SYNTAX por quartis** Quartil 1 (≤ 4.0) Quartil 2 (4.0-9.0) Quartil 3 (9.0-15.0) Quartil 4 (>15.0)	15 (20,0%) 15 (20,0%) 18 (24,0%) 27 (36,0%)	60 (26,7%) 59 (26,2%) 58 (25,8%) 48 (21,3%)	0,022^d^
**Escore SYNTAX por maior quartil** Baixo (≤15) Alto (>15)	48 (64,0%) 27 (36,0%)	177 (78,7%) 48 (21,3%)	0,011^a^

*IMC: Índice de massa corporal; LDL: lipoproteína de baixa densidade; HDL-C: lipoproteína de alta densidade; ALT: alanina aminotransferase; AST: aspartato aminotransferase; IIQ: intervalo interquartil; Quartil 1-4: quartil mais baixo para o mais alto; ^a^teste de qui-quadrado; ^b^teste t de amostras independentes; ^c^teste U de Mann-Whitney não paramétrico; ^d^teste de tendência de Cochran-Armitage.*

Os escores SYNTAX gerais variaram de 0 a 40, com mediana de 10 [IIQ (4,0–15,0)]. Em pacientes com câncer de pulmão, havia 20,0%; 20,0%; 24,0% e 36,0% dos pacientes nos quartis mais baixo, médio-inferior, médio-superior e mais alto, respectivamente. Nos pacientes controle, a porcentagem de pacientes foi de 26,7%; 26,2%; 25,8% e 21,3% do quartil mais baixo ao mais alto, respectivamente. O teste de tendência de Cochran-Armitage mostrou que a tendência de distribuição dos escores dos pacientes diferiu entre pacientes com câncer de pulmão e os controle, e o grupo com câncer tinham uma porcentagem significativamente mais alta de pacientes com escores SYNTAX mais alto. De acordo com a definição de escore SYNTAX alto e baixo pelo quartil mais alto (=15), assim como o escore como o valor de corte, 75 pacientes apresentaram escore alto (>15) e 225 pacientes apresentaram escore baixo (≤15). A taxa de escores altos foi maior em pacientes com câncer de pulmão do que nos pacientes controle ( Tabela 1 ).

### Associação entre a gravidade da DAC e o câncer de pulmão

A associação entre o escore SYNTAX e o câncer de pulmão foi analisada por quartis do escore. Associações significativas foram demonstradas entre o aumento do escore SYNTAX e o risco de desenvolver câncer de pulmão (P-trend=0,016). Após ajustes diferentes por idade, IMC, gênero e o hábito de fumar (os que nunca fumaram, os que já fumaram e os que fumam atualmente), o escore esteve correlacionado ao risco de câncer de pulmão (P-trend=0,024; P-trend=0,024; P-trend=0,029; P-trend=0,028, respectivamente). A razão de chances para câncer de pulmão (vs. o primeiro quartil do escore SYNTAX) foi 1.017 (IC95%: 0,457 a 2.265) para o segundo quartil, e 1.241 (IC95%: 0,572 a 2.693) para o terceiro quartil. O maior quartil do escore demonstrou risco significativamente maior de câncer de pulmão em comparação ao quartil mais baixo (OR: 2.250; IC95%: 1.077 a 4.699). Após ajuste por idade, IMC, gênero e hábito de fumar, os pacientes no maior quartil do escore tinham mais chances de desenvolver câncer de pulmão do que aqueles no quartil mais baixo do escore (OR: 2.149, IC95%: 1.008 a 4.584) ( Tabela 2 ).

**Tabela 2:** – OR e IC95% para câncer de pulmão por quartis do escore SYNTAX

	Quartis do Escore SYNTAX				
	Quartil 1 (Referência) (≤ 4,0)	Quartil 2 (4,0-9,0)	Quartil 3 (9,0-15,0)	Quartil 4 (>15,0)	*P* -trend^*^
Mediana do Escore Syntax	2,0	7,0	12,0	22,5	
Nº de câncer de pulmão / controles (Total)	15/60	15/59	18/58	27/48	
Modelo 1: OR (IC95%)	1	1,017 (0,457-2,265)	1,241 (0,572-2,693)	2,250 (1,077-4,699)	0,016
Modelo 2: OR (IC95%)	1	1,007 (0,451-2,245)	1,233 (0,568-2,677)	2,151 (1,021-4,530)	0,024
Modelo 3: OR (IC95%)	1	1,011 (0,453-2,258)	1,230 (0,566-2,672)	2,165 (1,024-4,545)	0,024
Modelo 4: OR (IC95%)	1	1,010 (0,452-2,255)	1,215 (0,557-2,653)	2,133(1,002-4,540)	0,029
Modelo 5: OR (IC95%)	1	1,019 (0,455-2,279)	1,227 (0,561-2,285)	2,149 (1,008-4,584)	0,028

Modelo 1: modelo bruto; Modelo 2: ajustado por idade; Modelo 3: ajustado por idade, IMC; Modelo 4: ajustado por idade, IMC, gênero; Modelo 5: ajustado por idade, IMC, gênero, hábito de fumar (nunca fumaram, já fumaram e fumam atualmente). Quartis 1-4: do mais baixo para o mais alto. *Teste de tendência com base em variáveis contendo valor mediano para cada quartil.

Depois, a análise de regressão logística mostrou mais riscos de escore alto para câncer de pulmão em comparação ao escore baixo. A análise univariada de regressão logística mostrou que o escore alto aumentou o risco de câncer de pulmão em 2.074 vezes (IC95%: 1.174-3.665). Ajustando por idade, a análise multivariada de regressão logística mostrou que pacientes com escore alto tinham 1.994 mais chances de desenvolver câncer de pulmão (IC95%: 1.119-3.551). Depois, ajustando por idade, IMC, gênero e hábito de fumar (os que nunca fumaram, os que já fumaram e os que fumam atualmente), pacientes com escore alto tinham 1.985 mais chances de desenvolver câncer de pulmão (IC95%: 1.105-3.563) em comparação aos pacientes com escore baixo ( Tabela 3 ).

**Tabela 3:** – OR e IC95% do escore SYNTAX alto para câncer de pulmão

Modelo	OR	IC95%	Valor de p
Modelo 1	2,074	1,174-3,665	0,012
Modelo 2	1,994	1,119-3,551	0,019
Modelo 3	2,007	1,123-3,588	0,019
Modelo 4	1,983	1,105-3,558	0,022
Modelo 5	1,985	1,105-3,563	0,022

*Modelo 1: modelo bruto; Modelo 2: ajustado por idade; Modelo 3: ajustado por idade, IMC; Modelo 4: ajustado por idade, IMC, gênero; Modelo 5: ajustado por idade, IMC, gênero, hábito de fumar (nunca fumaram, já fumaram e fumam atualmente).*

## Discussão

Tanto a DAC quanto o câncer de pulmão são doenças que costumam afetar a saúde do ser humano. A doença isquêmica do coração padronizada por idade e o câncer de pulmão foram a segunda e a terceira principais causas de anos potenciais perdidos de vida na China, em 2017.^16^ A DAC raramente está associada ao câncer de pulmão recém-diagnosticado. Até onde sabemos, este estudo é o primeiro a investigar a associação direta entre a gravidade anatômica da DAC e o câncer de pulmão. Os resultados demonstraram que a gravidade da DAC está associada ao risco aumentado de câncer de pulmão. Após diferentes ajustes, a DAC grave ainda esteve significativamente associada ao risco de câncer de pulmão. Dada a importância da DAC e do câncer de pulmão na saúde, os resultados deste estudo são extremamente significativos.

A cardio-oncologia é um campo médico emergente que foca na cardiotoxicidade induzida pelo tratamento anticâncer.^3 , 17 , 18^ Porém, a relação direta entre o câncer e a doença cardíaca ainda é amplamente desconhecida. Neste estudo, avaliamos a gravidade da DAC utilizando o escore SYNTAX com base nos angiogramas coronários de pacientes com câncer de pulmão e controle. Os resultados demonstraram que havia associações significativas entre o aumento do escore e o risco de câncer de pulmão. A DAC e o câncer de pulmão são considerados grandes problemas de saúde pública, crescendo em importância em termos globais. Os resultados deste estudo demonstraram que pacientes com DAC e escore SYNTAX alto tinham mais riscos de desenvolver câncer de pulmão em comparação a pacientes com escore baixo, o que indica que é importante fazer a triagem de câncer de pulmão em pacientes com DAC grave. Deve-se mencionar que o fato de ambas as doenças estarem em uma relação de causa e efeito, ou de que elas coexistam em um ambiente comum, não pôde ser determinado neste estudo. É necessário esclarecer melhor a correção entre a DAC e o câncer de pulmão em análises futuras.

O mecanismo por trás da associação entre a DAC e o câncer não é bem conhecido, e isso pode se dever a vários aspectos: fatores de risco compartilhados, status inflamatório e vias fisiopatológicas compartilhadas.^9 , 10 , 19^ Este estudo mostra que a associação entre a DAC e o câncer de pulmão é independente dos fatores de risco cardiovasculares e do câncer, incluindo idade, IMC, gênero e o hábito de fumar. Muitos estudos demonstraram que a inflamação impacta a patogênese do câncer e da DAC.^20 - 23^ Dados clínicos e experimentais têm papel essencial na inflamação da aterotrombose, incluindo a DAC.^24^ As inflamações relacionadas ao câncer podem estar presentes antes que ocorra uma mudança maligna, o que pode levar ao desenvolvimento de tumores.^20^ Foi reportado que ratos com tumores têm uma resposta pró-inflamatória robusta aos tumores.^25 , 26^ O estudo Canakinumab Anti-inflammatory Thrombosis Outcomes Study (CANTOS) mostra que o tratamento anti-inflamatório com Canaquinumabe pode reduzir significativamente a incidência de câncer de pulmão e sua mortalidade, além de diminuir as taxas de eventos cardiovasculares recorrentes entre pacientes com infarto do miocárdio prévio.^24 , 27^ Com base em estudos anteriores, é razoável inferir que a inflamação traz uma relação considerável entre a DAC e o câncer de pulmão. A proteína C-reativa é o marcador inflamatório mais aceito.^21 , 28^ No estudo retrospectivo, mais de um terço dos pacientes não tinham sido testados para a proteína C-reativa, então não pudemos analisar o papel da inflamação na associação entre a DAC e o câncer de pulmão neste estudo. Vamos determinar o impacto da inflamação na relação direta entre a DAC e o câncer em novas análises.

Além da inflamação, a doença aterotrombótica e os neoplasmas compartilham muitas vias fisiopatológicas, incluindo estresse oxidativo, apoptose, proliferação celular e neoangiogênese.^19^ As micropartículas de RNA foram um grande mecanismo fisiopatológico entre o câncer e a aterosclerose.^19^ O receptor 1 de Lipoproteína de Baixa Densidade Oxidada (LOX-1) é um receptor para lipoproteínas de baixa densidade oxidadas (ox-LDL) primariamente expressas nas células endoteliais e nos órgãos ricos em vasos sanguíneos. O LOX-1 demonstrou ser efetivo durante o processo de aterogênese e tumorigênese, e poderia ser uma conexão importante entre essas duas doenças. Indivíduos com placas ateroscleróticas e altos níveis de expressão de ox-LDL e LOX-1 parecem ser mais suscetíveis a desenvolver câncer.^29^ A associação entre DAC e câncer pode ser explicada pela sobreposição mecânica na fisiopatologia da aterogênese e tumorigênese; porém, mais estudos são necessários para esclarecer o mecanismo por trás da associação entre a DAC e o câncer de pulmão.

### Limitações do estudo

As limitações incluem, primeiramente, o pequeno tamanho da amostra. Resultados de uma população específica e pequena de pacientes de um único centro da China podem ser divergentes. Porém, o viés da inclusão no estudo foi minimizado com base na escolha dos pacientes. Incluímos todos os pacientes elegíveis com câncer de pulmão que não tinham recebido tratamentos anticâncer, e incluímos aleatoriamente os pacientes controle, com base nos escores de propensão, dentre 49.141 pacientes.

Em segundo lugar, o mecanismo por trás da associação entre a DAC e o câncer de pulmão não ficou claro, embora a inflamação possa ter um papel importante para o risco aumentado de DAC grave e câncer de pulmão. Vamos avaliar mais a associação entre DAC e câncer e o mecanismo por trás disso com base no estudo piloto.

## Conclusões

Este estudo promove uma nova perspectiva na relação entre a DAC e o câncer de pulmão. Demonstramos que a gravidade anatômica da DAC e o câncer de pulmão estão associados ao risco do câncer de pulmão. Os resultados nos alertam de que possa valer a pena fazer uma triagem mais detalhada de câncer de pulmão em pacientes com DAC grave. Vale ilustrar a conexão direta entre a DAC e o câncer de pulmão e esclarecer o mecanismo deste processo no futuro, em ensaios clínicos de larga escala e estudos básicos.
