# Supplementary material for: Associação entre a Gravidade da Doença Arterial Coronariana e Câncer de Pulmão: Um Estudo Piloto Transversal
Source: Arq Bras Cardiol. 2021 Dec 16;118(2):478–85. [Article in Portuguese] doi: 10.36660/abc.20200478 (PMC8856686; doi:10.36660/abc.20200478)
Supplement: Translation

# Translation: Association between the Severity of Coronary Artery Disease and Lung Cancer: A Pilot Cross-Sectional Study

**Authors:** Mingzhuang Sun, Qian Yang, Meng Li, Jing Jing, Hao Zhou, Yundai Chen, Shunying Hu

**Affiliations:** 1 China Chinese PLA General Hospital - Department of Cardiology, Beijing – China; 2 China Chinese PLA General Hospital - Department of Medical Records Management, Beijing – China

**Keywords:** Coronary Artery Disease/complications, Lung Neoplasms/complications, Coronary Angiography/methods, Severity of Illness Index, Percutaneous Coronary Intervention/methods

## Abstract

**Background:**

The direct relationship between coronary artery disease (CAD) and lung cancer is not well known.

**Objective:**

To investigate the association between the anatomical severity of CAD and lung cancer.

**Methods:**

Three-hundred study patients, including 75 recently diagnosed lung cancer patients and 225 matched non-cancer patients, underwent coronary angiography during hospitalization without previous percutaneous coronary intervention (PCI) or coronary artery bypass grafting (CABG). The SYNTAX score (SXscore) was used to assess the severity of CAD. A high SXscore (SXhigh) grade was defined as SXscore > 15 (the highest quartile of the SXscore). The Cochran-Armitage test for trend was used to assess the distribution of patients’ SXscores. Logistic regression analysis was used to assess the association between the severity of CAD and lung cancer. P-values were set when significance level was 5%.

**Results:**

The distribution trend of patients’ SXscore by quartiles was different between lung cancer patients and control patients (from the lowest to the highest quartile: 20.0%, 20.0%, 24.0%, 36.0% vs. 26.7%, 26.2%, 25.8%, 21.3%, p=0.022). The SX high rate was higher in lung cancer patients than in control patients (36.0% vs. 21.3%, p=0.011).The highest quartile of the SXscore showed higher risk of lung cancer in comparison to the lowest quartile (OR: 2,250, 95%CI: 1,077 to 4,699 ; P-trend= 0.016). After adjustment, patients in the highest quartile of the SXscore had higher risk of lung cancer (OR: 2,149, 95%CI: 1,008 to 4,584; P-trend= 0.028). Patients with high SXscore (> 15) had 1,985 times more chances of having lung cancer (95%CI: 1,105–3,563, P= 0.022).

**Conclusions:**

The anatomical severity of CAD is associated with the risk of lung cancer, which indicates that a thorough lung cancer screening may be significant among severe CAD patients.

## Introduction

Both cancer and heart diseases are critical health problems for human beings worldwide.^1 , 2^ Anticancer therapy-induced cardiovascular toxicity has led to the new interdisciplinary field of cardio-oncology.^2 - 5^ At present, oncologists and cardiologists mostly pay more attention to anticancer therapy-related heart diseases among cancer survivors.^6 , 7^ Some studies show there is a direct interactional relationship between heart diseases and cancer.^8^

Coronary artery disease (CAD) and cancer share common risk factors and pathophysiological mechanisms.^9 - 11^ There is increasing evidence showing that cancer patients were at increased risk of cardiovascular diseases. A Swedish study showed that cancers were associated with an increased risk of CAD; however, in that case, the study patients had been administered anticancer therapies, so the study could not demonstrate whether or not there was a direct association between CAD and cancer.^2^ Whether or not the severity of CAD is directly associated with cancer has been rarely reported until now. It is essential to elucidate if there is a potential direct link between CAD and cancer in order to provide better understanding and better management for these two important diseases.

Lung cancer is the most common cancer and the leading cause of cancer death.^12^ It is worth investigating the association between CAD and lung cancer. A meta-analysis showed that lung cancer was associated with significantly increased risk of CAD during follow-up compared with non-lung cancer.^13^ In the study, the included lung cancer patients were followed-up for more than one year after being diagnosed, so they were more likely to have received anticancer therapies, which could not exclude the effect of anticancer treatments on CAD. The direct relationship between CAD and lung cancer is not well understood until the present time. The hypothesis that the severity of CAD is associated with lung cancer has not been reported.

We conducted a cross-sectional study to investigate the direct association between the anatomical severity of CAD and lung cancer. All of the lung cancer patients had been recently diagnosed and did not receive any anticancer treatments. The control patients were non-lung cancer patients enrolled by propensity score matching to lung cancer patients. All of the study patients underwent coronary angiography (CAG) during index hospitalization. The anatomical severity of CAD was assessed using the SYNTAX score (SXscore) based on coronary angiograms.^14^ The objective of the present study was to determine whether or not the anatomical severity of CAD is related to the risk of lung cancer.

## Patients and Methods

### Study patients

In Chinese PLA General Hospital, all data of inpatients are stored in the medical record system, including coronary angiograms. There were 173 patients with lung cancer (ICD-10 code 34), and 48,968 without any cancers who underwent CAG (ICD-9-CM codes 88.5, 88.55, 88.56, and 88.57) in the department of cardiology from January 1st, 2009, to July 31, 2019.

Lung cancer diagnoses were validated according to the pathological diagnosis. Of the 173 lung cancer patients, 75 who had been recently diagnosed without being administered any anticancer treatments were enrolled in this study, excluding 31 with a history of anticancer therapy and 67 who were previously undergoing percutaneous coronary intervention (PCI) or coronary artery bypass grafting (CABG). Among the 48,968 non-cancer patients, 225 were enrolled as control patients by propensity score matching to the lung cancer patients (1:3 ratio), according to age, gender, family history of CAD, body mass index (BMI), smoking, hypertension, diabetes, and hyperlipidemia. None of the included patients had a history of connective tissue or other inflammatory diseases, nor of PCI or CABG before the index hospitalization. A flowchart of the study patient enrollment process is shown in Figure 1 .

**Figure 1:** – Flowchart of study patients.

We calculated sample size using the PASS software. According to parameters including a= 0.05, b = 0.20, OR= 2 and the case-control ratio= 1:3, the sample size of the lung cancer group was 74, and the sample size of the non-lung cancer group was 222. In this study, we enrolled 300 patients (75 lung cancer patients and 225 non-lung cancer patients) who met the sample size requirement.

### SXscores of CAD based on CAG

The severity of CAD was evaluated using the SXscore algorithm (described in full elsewhere).^14 , 15^ All of the angiographic variables related to the SXscore calculation were computed by two blinded experienced interventional cardiologists. When the SXscore of each patient was different between the two cardiologists, they would discuss the angiograms and reach a common SXscore. Final SXscores were calculated per patient and saved in a dedicated database. The SXscore of 15 was the highest quartile in the study. A low SXscore (SXlow) was defined as SXscore ≤15, and a high SXscore (SXhigh) as SXscore >15. In the logistic regression analysis, we defined SXhigh (> 15) as positive.

### Statistical analysis

Descriptive statistics are presented as frequency and percentage rates for categorical variables, and mean ± standard deviation (SD) and medians [interquartile range (IQR)] for continuous variables, according to normality of the data. We assessed the normality of the data using the Skewness and Kurtosis normality tests. We used Independent-Samples t-test to compare means between groups when variables were normally distributed. The Mann-Whitney U non-parametric statistical test was used for continuous variables without normal distribution, including triglycerides, low-density lipoprotein cholesterol, high density lipoprotein cholesterol, fasting blood-glucose, alanine aminotransferase, aspartate aminotransferase, serum creatinine and uric acid. The chi-square or Fisher exact tests were used to examine differences for categorical measures.

The SXscore was divided into quartiles based on the distribution of the score in all of the study subjects, being the first quartile used as a reference. SXscore values for quartiles 1, 2, 3, and 4 were *<* 4.0, 4.0–9.0, 9.0–15.0, and *>* 15.0, respectively. The Cochran-Armitage test for trend was used to assess the distribution of the patients’ SXscores. We assessed the relationship between CAD severity (SXscore stratified by quartiles) and lung cancer by using the logistic-regression analysis, adjusting for common related risk factors, including age, BMI, gender, and smoking. Odds ratio (OR) and 95% confidence intervals (CI) were calculated. P-values were two-tailed, and we set the level of significance at 5%. Statistical analyses were performed using the SPSS software, version 23 (SPSS, Inc. Chicago, IL, USA), and the SAS software (SAS Institute, Cary, Carolina do Norte).

## Results

### Patients’ characteristics

The study patients were a specific population from the Chinese PLA general hospital. The mean age of the 300 study patients was 63.5±9.7 years, and 70 (23.3%) were female. All of the included 75 lung cancer patients had pathological evidence of cancer; 69 (92%) were diagnosed with non-small cell lung cancer; and the other 6 patients (8%), with small cell lung cancer. Forty-eight patients (75%) were at stage I or II; 12 patients (16%) were at stage III; 4 patients (5.3%) were at stage IV; and it was not possible to confirm the disease stage for the other 11 patients (14.7%). Four patients (5.3%) were metastatic. Lung cancer patients had not received any anticancer treatments before the index hospitalization.

There was no significant difference for age, gender, smoking (stratified as never-smokers and ever-smokers), BMI, family history of CAD, hypertension, diabetes, and hyperlipidemia between lung cancer patients and non-lung cancer patients. However, history of smoking is different between the lung cancer group and the control groups when history of smoking was stratified as never-smokers, former-smokers and current-smokers, which was adjusted in the logistic regression analysis. Ejection fraction (EF) and laboratory data were comparable between lung cancer patients and control patients ( Table 1 ).

**Table 1:** – Patients’ characteristics

Characteristic	Lung cancer group (n = 75)	Non-cancer group (n = 225)	p-value
**Gender, n (%)** Male Female	59 (78.7%) 16 (21.3%)	171 (76.0%) 54 (24.0%)	0.636^a^
**Age (years)** <65 ≥65	34(45.3%) 41(54.7%)	120 (53.3%) 105 (46.7%)	0.230^a^
**BMI, n (%)** ≤24 >24	25 (33.30%) 50 (66.70%)	73 (32.4%) 152 (67.6%)	0.887^a^
**Smoking, n (%)** Never-smokers Ever-smokers	27(36.0%) 48(64.0%)	97 (43.1%) 128 (56.9%)	0.279^a^
**Smoking, n (%)** Never-smokers Former-smokers Current-smokers	27 (36.0%) 21 (28.0%) 27 (36.0%)	97 (43.1%) 30 (13.3%) 98 (43.6%)	0.014^a^
**Family history of CAD, n (%)** No Yes	69 (92.00%) 6 (8.00%)	196 (87.1%) 29 (12.9%)	0.253^a^
**Hypertension, n (%)** No Yes	29 (38.7%) 46 (61.3%)	84 (37.3%) 141 (62.7%)	0.836^a^
**Diabetes, n (%)** No Yes	40 (53.3%) 35 (46.7%)	131 (58.2%) 94 (41.8%)	0.459^a^
**Hyperlipidemia, n (%)** No Yes	25(33.3%) 50(66.7%)	95 (42.2%) 130 (57.8%)	0.174^a^
**Ejection fraction (%), n (%)** <50 ≥50	5(7.0%) 66(93.0%)	21 (11.7%) 159 (88.3%)	0.279^a^
**Laboratory data** Total cholesterol Triglyceride, mmol/L LDL-C , mmol/L HDL-C, mmol/L FBG, mmol/L ALT, U/L AST, U/L Albumin, g/L Serum creatinine, µmol/L Uric acid, mmol/L Hemoglobin, g/L	4.035±0.950 1.420 (IQR [1.055–1.780]) 2.545 (IQR [1.838–3.195]) 1.070 (IQR [0.833–1.265]) 5.820 (IQR [4.980–7.720]) 20.50 (IQR [11.70–27.20]) 17.20 (IQR [13.70–25.10]) 40.589±4.056 76.70 (IQR [66.80–84.80]) 328.10 (IQR [266.00–396.70]) 135.76±16.647	4.029±1.015 1.360 (IQR [1.020–1.860]) 2.340 (IQR [1.850–3.058]) 0.980 (IQR [0.830–1185]) 5.710 (IQR [4.895–7.725]) 21.40 (IQR [15.20–30.70]) 18.35 (IQR [14.70–24.55]) 40.640±6.459 75.50 (IQR [64.90–89.25]) 324.95 (IQR [268.28–358.13]) 139.55±16.059	0.968^b^ 0.854^c^ 0.497^c^ 0.096^c^ 0.598^c^ 0.049^c^ 0.355^c^ 0.949^b^ 0.910^c^ 0.984^c^ 0.088^b^
**SXscore grades by quartiles** Quartile 1 (≤ 4.0) Quartile 2 (4.0-9.0) Quartile 3 (9.0-15.0) Quartile 4 (>15.0)	15 (20.0%) 15 (20.0%) 18 (24.0%) 27 (36.0%)	60 (26.7%) 59 (26.2%) 58 (25.8%) 48 (21.3%)	0.022^d^
**SXscore grades by highest quartile** SXlow (≤15) SXhigh (>15)	48 (64.0%) 27 (36.0%)	177 (78.7%) 48 (21.3%)	0.011^a^

*BMI: body mass index; LDL-C:low-density lipoprotein cholesterol; HDL-C: high density lipoprotein cholesterol; FBG: fasting blood-glucose; ALT: alanine aminotransferase; AST: aspartate aminotransferase; IQR: interquartile range; Quartile1-4 = lowest quartile to highest quartile;^a^Chi-squared test;^b^Independent-Samples t test;^c^Mann-Whitney U non-parametric statistical test;^d^Cochran–Armitage trend test.*

Overall SXscores ranged from 0 to 40, with a median of 10 [IQR (4.0–15.0)]. In lung cancer patients, there were 20.0%, 20.0%, 24.0%, and 36.0% of patients in the lowest, lower-middle, upper-middle, and highest quartile, respectively. In control patients, the percentage of patients was 26.7%, 26.2%, 25.8%, and 21.3% from the lowest to the highest quartile, respectively. The Cochran-Armitage test for trend showed that the distribution trend of the patients’ SXscore was different between lung cancer and control patients, and the lung cancer group had a significantly higher percentage of patients with higher SXscore. According to the definition of SXhigh and SXlow by the highest quartile (SXscore=15), of SXscores as the cut-off value, 75 patients presented with SXhigh (>15), and 225 patients with SXlow (≤15). The rate of SXhigh was higher in lung cancer patients than in control patients ( Table 1 ).

### Association between severity of CAD and lung cancer

The association between the SXscore and lung cancer was analyzed by quartiles of SXscore. Significant associations were shown between the increasing SXscore and the risk of lung cancer (P-trend=0.016). After different adjustments for age, BMI, gender and smoking (never smokers, former smokers and current smokers), the SXscore was correlated with the risk of lung cancer (P-trend=0.024; P-trend=0.024; P-trend=0.029; P-trend=0.028, respectively). The OR for lung cancer (vs. the first quartile of the SXscore) was 1,017 (95%CI: 0,457 to 2,265) for the second quartile of SXscore, and was 1.241 (95% CI: 0.572 to 2.693) for the third quartile of the SXscore. The highest quartile of the SXscore showed significantly higher risk of lung cancer compared to the lowest quartile (OR: 2.250; 95%CI: 1,077 to 4,699). After adjusting for age, BMI, gender and smoking, patients in the highest quartile of the SXscore had higher risk of lung cancer than those in the lowest quartile of the SXscore (OR: 2,149, 95%CI: 1,008 to 4,584 ) ( Table 2 ).

**Table 2:** – ORs and 95%CIs for lung cancer by quartiles of the SXscore

	Quartile of SXscore				
	Quartile 1 (Reference) (≤ 4.0)	Quartile 2 (4.0-9.0)	Quartile 3 (9.0-15.0)	Quartile 4 (>15.0)	*P* for trend^*^
Median of SXscore	2.0	7.0	12.0	22.5	
No. of lung cancer/control (Total)	15/60	15/59	18/58	27/48	
Model 1: OR (95% CI)	1	1.017 (0.457-2.265)	1.241 (0.572-2.693)	2.250 (1.077-4.699)	0.016
Model 2: OR (95% CI)	1	1.007 (0.451-2.245)	1.233 (0.568-2.677)	2.151 (1.021-4.530)	0.024
Model 3: OR (95% CI)	1	1.011 (0.453-2.258)	1.230 (0.566-2.672)	2.165 (1.024-4.545)	0.024
Model 4: OR (95% CI)	1	1.010 (0.452-2.255)	1.215 (0.557-2.653)	2.133(1.002-4.540)	0.029
Model 5: OR (95% CI)	1	1.019 (0.455-2.279)	1.227 (0.561-2.285)	2.149 (1.008-4.584)	0.028

Model 1: crude model; Model 2: adjusted for age; Model 3: adjusted for age, BMI; Model 4: adjusted for age, BMI, gender; Model 5: adjusted for age, BMI, gender, smoking (never smokers, former smokers and current smokers). Quartile 1-4 = lowest quartile to highest quartile. *Test for trend based on variable containing median value for each quartile.

Then, a logistic regression analysis showed higher risk of SXhigh for lung cancer compared with SXlow. Univariate logistic regression analysis showed that SXhigh increased the risk of lung cancer by 2,074 times (95%CI: 1,174–3,665). Adjusting for age, the multivariate logistic regression analysis showed that patients with SXhigh had 1,994 more chances of developing lung cancer (95%CI: 1,119–3,551). Then, adjusting for age, BMI, gender and smoking (never smokers, former smokers and current smokers), patients with SXhigh were more likely to have lung cancer by 1,985 times (95%CI: 1,105–3,563) compared to patients with SXlow ( Table 3 ).

**Table 3:** – ORs and 95%CIs of SXhigh for lung cancer

Model	OR	95%CI	p-value
Model 1	2.074	1.174-3.665	0.012
Model 2	1.994	1.119-3.551	0.019
Model 3	2.007	1.123-3.588	0.019
Model 4	1.983	1.105-3.558	0.022
Model 5	1.985	1.105-3.563	0.022

*Model 1: crude model; Model 2: adjusted for age; Model 3: adjusted for age, BMI; Model 4: adjusted for age, BMI, gender; Model 5: adjusted for age, BMI, gender, smoking (never smokers, former smokers and current smokers).*

## Discussion

Both CAD and lung cancer are diseases that usually affect human health. Age standardized ischemic heart disease and lung cancer were the second and third leading causes of years of life lost in China in 2017.^16^ CAD is rarely directly associated with recently-diagnosed lung cancer. To the best of our knowledge, the current study is the first to investigate the direct association between the anatomical severity of CAD and lung cancer. The results showed that the severity of CAD is associated with increased risk of lung cancer. After different adjustments, severe CAD was still significantly associated with the risk of lung cancer. Given the importance of CAD and lung cancer to human health, the results of this study are of great significance.

Cardio-oncology is an emerging medical field that focuses on cardiotoxicity induced by anticancer therapy.^3 , 17 , 18^ However, the direct relationship between cancer and heart disease remains largely unknown. In this study, we evaluated the severity of CAD using the SXscore based on coronary angiograms of lung cancer patients and non-lung cancer patients. The results showed there were significant associations between increasing SXscore and the risk of lung cancer. CAD and lung cancer are considered as significant public health problems, growing in importance, globally. The results of the present study demonstrated that CAD patients with high SXscore were at higher risk of lung cancer compared with patients with low SXscore, which indicates that it would be preferable to screen lung cancer among severe CAD patients. It should be pointed out that the fact that both diseases are in a cause-effect relationship or that they coexist in a common environment could not be determined in the current study. It is necessary to further clarify the correlation between CAD and lung cancer in future analyses.

The mechanism underlying the association between CAD and cancer is not well known, and that may be owed to several possible aspects: shared risk factors, inflammatory status and shared pathophysiological pathways.^9 , 10 , 19^ This study shows that the association between CAD and lung cancer is independent from common cardiovascular and cancer risk factors, including age, BMI, gender and smoking. A great number of studies showed that inflammation plays a key role in the pathogenesis of cancer and CAD.^20 - 23^ Clinical and experimental data support a critical role for inflammation in atherothrombosis, including CAD.^24^ Cancer-related inflammations can be present before a malignant change occurs, which can lead to the development of tumors.^20^ It has been reported that tumor-bearing mice have a robust proinflammatory response to tumors.^25 , 26^ The Canakinumab Anti-inflammatory Thrombosis Outcomes Study (CANTOS) shows that anti-inflammatory therapy with Canakinumab could significantly reduce the incidence of lung cancer and lung cancer mortality, in addition to significantly reducing the rate of recurrent cardiovascular events among patients with previous myocardial infarction.^24 , 27^ Based on previous studies, it is reasonable to infer that inflammation is an important link between CAD and lung cancer. The C-reactive protein is the most extensively accepted inflammatory marker.^21 , 28^ In the retrospective study, more than one-third of the study patients had not been tested for C-reactive protein, so we could not further analyze the role of inflammation in the association between CAD and lung cancer in the present study. We will determine the impact of inflammation on the direct relationship between CAD and cancer in future studies.

Apart from inflammation, atherothrombotic disease and neoplasms share many pathophysiologic pathways, including oxidative stress, apoptosis, Cell proliferation, and neoangiogenesis.^19^ RNA microparticles were a major pathophysiologic mechanism between cancer and atherosclerosis.^19^ Lectin-like oxidized low-density lipoprotein receptor-1 (LOX-1) is a lectin-like receptor for oxidized low-density lipoproteins (ox-LDL) primarily expressed in endothelial cells and blood vessel rich organs. LOX-1 has demonstrated to be effective during the process of atherogenesis and tumorigenesis, and could be a potential link between these two diseases. Individuals with atherosclerotic plaques and high levels of circulating ox-LDL and LOX-1 expression seem to be more prone to developing cancer.^29^ The association of CAD and cancer can be explained by the mechanistic overlap in the pathophysiology of atherogenesis and tumorigenesis; however, more studies are necessary to further elucidate the mechanism underlying the association between CAD and cancer.

### Study limitations

The limitations include, firstly, the small sample size. Results from a specific small-sized population of patients from a single center of China might be deviant. However, the enrollment bias was as minimized as possible based on the choice of study patients. We enrolled all eligible lung cancer patients who had not received any anticancer treatments, and randomly enrolled propensity score matched control patients among 49,141 patients. Secondly, the underlying mechanism of the association between CAD and lung cancer was not clarified, although inflammation may play an important role for the increased risk of severe CAD with lung cancer. We will further investigate the association between CAD and cancer and the underlying mechanism on the basis of the pilot study.

## Conclusions

This study provides a new perspective on the relationship between CAD and lung cancer. We demonstrate that the anatomical severity of CAD and lung cancer is associated with the risk of lung cancer. The results warns us that it may be worth it to closely screen lung cancer among patients with severe CAD. It is significant to further illustrate the direct link between CAD and lung cancer and to elucidate the underlying mechanism in future, larger-scale clinical trials and basic studies.
